# Valorization of Seafood Waste for Food Packaging Development

**DOI:** 10.3390/foods13132122

**Published:** 2024-07-03

**Authors:** Zhijing Zhan, Yiming Feng, Jikai Zhao, Mingyu Qiao, Qing Jin

**Affiliations:** 1 School of Food and Agriculture, University of Maine, Orono, ME 04469, USA; zhijing.zhan@maine.edu; 2 Virginia Seafood AREC, Virginia Polytechnic Institute and State University, Hampton, VA 23662, USA; yimingfeng@vt.edu; 3 Department of Biological Systems Engineering, Virginia Polytechnic Institute and State University, Blacksburg, VA 24061, USA; 4 School of Earth, Environmental, and Marine Sciences, The University of Texas Rio Grande Valley, Edinburg, TX 78542, USA; jikai.zhao@utrgv.edu; 5 Department of Nutritional Sciences, University of Connecticut, Storrs, CT 06269, USA; mingyu.qiao@uconn.edu; 6 Center for Clean Energy Engineering (C2E2), University of Connecticut, Storrs, CT 05269, USA; 7 Institute of Materials Science (IMS), University of Connecticut, Storrs, CT 06269, USA

**Keywords:** seafood waste, biopolymers, nanomaterials, biodegradable packaging

## Abstract

Packaging plays a crucial role in protecting food by providing excellent mechanical properties as well as effectively blocking water vapor, oxygen, oil, and other contaminants. The low degradation of widely used petroleum-based plastics leads to environmental pollution and poses health risks. This has drawn interest in renewable biopolymers as sustainable alternatives. The seafood industry generates significant waste that is rich in bioactive substances like chitin, chitosan, gelatins, and alginate, which can replace synthetic polymers in food packaging. Although biopolymers offer biodegradability, biocompatibility, and non-toxicity, their films often lack mechanical and barrier properties compared with synthetic polymer films. This comprehensive review discusses the chemical structure, characteristics, and extraction methods of biopolymers derived from seafood waste and their usage in the packaging area as reinforcement or base materials to guide researchers toward successful plastics replacement and commercialization. Our review highlights recent advancements in improving the thermal durability, mechanical strength, and barrier properties of seafood waste-derived packaging, explores the mechanisms behind these improvements, and briefly mentions the antimicrobial activities and mechanisms gained from these biopolymers. In addition, the remaining challenges and future directions for using seafood waste-derived biopolymers for packaging are discussed. This review aims to guide ongoing efforts to develop seafood waste-derived biopolymer films that can ultimately replace traditional plastic packaging.

## 1. Introduction

Globally, approximately 178 million tons of seafood, including fish, shellfish, shrimp, lobster, crab, and algae, were harvested in 2020 and were estimated to be valued at $200 billion [1]. Seafood products are nutritionally dense, including high contents of protein, omega-3 fatty acids, selenium, and iodine, which are difficult to find in other foods. Seafood also requires less farming, freshwater, land, processing, and carbon emissions to produce, which makes it more environmentally friendly than other high-protein foods, such as those from terrestrially farmed animals [2]. In recent years, seafood has become essential in global diets, providing 20% of animal protein for over 3 billion people in the world. Furthermore, global seafood production is projected to increase by over 13% in value by 2030. In contrast, approximately 30% to 35% of seafood is wasted globally [1].

The modern seafood processing industry generates a large volume of waste, including heads, tails, shells, backbones, etc. Waste amounts vary depending on the type of seafood. The waste from crustaceans such as crabs and lobsters is primarily composed of shells and cephalothoraxes, which constitute approximately 30–40% of their overall fresh weight [3]. The waste from fish constitutes 25–60% of their total wet weight and is mainly composed of heads, skin, scales, and bones [4]. Normally, these waste products end up being buried, landfilled, dumped into the sea, or, even worse, left to spoil [5], all of which have adverse consequences for both public health and the ecosystem. For example, dumping seafood waste into the sea can lead to the entombment or suffocation of marine life, as well as the introduction of diseases and non-native invasive species [6]. Furthermore, Xu et al. reported that food waste previously landfilled can generate methane (CH_4_), ammonia (NH_3_), and hydrogen sulfide (H_2_S) [7], which are competing with and reducing agricultural land and are also harmful to the environment.

These waste products are still useful because they usually contain high-value components such as alginate, protein, chitin, gelatin, minerals, fatty acids, and peptides [8]. These components possess immense potential for repurposing across diverse sectors such as pharmaceuticals, the food industry, and cosmetics [9]. Moreover, some marine biopolymers have shown great potential as substitutes for artificial polymers. These natural biopolymers could therefore be used in the creation of eco-friendly and sustainable solutions in food packaging, medical drug packaging, and tissue replacement due to their biocompatibility and biodegradability [10].

In the contemporary food sector, packaging serves the crucial function of safeguarding edibles from harm and augmenting their storage duration. Traditionally, petroleum-based plastics such as polyvinylchloride [11], polyethylene terephthalate [12], polystyrene [13], and polyamide [14] are widely used in food packaging due to their affordability, superior mechanical properties, and exceptional barrier characteristics. However, with undesirable environmental effects and concerns about the lack of fossil energies [15], the industry is looking for biodegradable materials with the same functional properties to replace these petroleum-based materials in food packaging. In this context, the utilization of marine waste for the production of eco-friendly food biopolymers has been investigated to create compostable packaging solutions [9].

**Figure 1:**
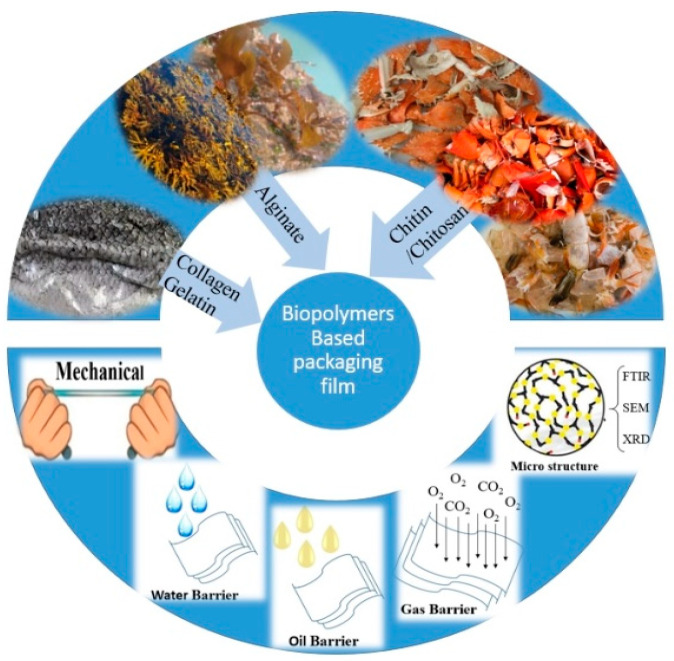
Biopolymers derived from seafood waste with potential food packaging applications.

Many biopolymers derived from seafood waste show promise for use as food packaging materials (Figure 1). For example, chitin could be derived from the outer skeletons of arthropods, such as lobster shells [16]. Chitosan, which is the deacetylated form of chitin, can also be harvested from such arthropods [17]. Gelatin, derived from marine waste such as fish skin and scales, is a renewable and biodegradable material that has been proven to form transparent and flexible films [18]. Alginate, a biocompatible and biodegradable biopolymer, can be extracted from waste seaweed [19,20,21]. Nanomaterials such as nanochitin and nanochitosan can be derived from corresponding chitin and chitosan.

Biopolymers from seafood waste offer significant potential for food packaging applications, reducing reliance on petroleum-based plastics and supporting environmental conservation. While biopolymers possess commendable attributes that make them suitable for food packaging, opportunities for improvement remain in enhancing their mechanical, thermal, and barrier properties [17,22,23]. This article reviews contemporary studies (2016–2024) on the utilization of seafood byproducts to produce biopolymers for food packaging. Biopolymers, including chitin, chitosan, alginate, gelatin, and nanoparticles derived from seafood waste, are discussed in detail. We focused on their structures, properties, production methods, and recent applications in food packaging. We summarized and evaluated advancements in biopolymer films and highlighted key principles and breakthroughs in their development. Converting seafood waste to biopolymers can significantly reduce waste generation and associated processing costs, improving the economic and environmental viability of the entire seafood industry. Furthermore, using the produced biopolymers as food packaging materials can serve as an alternative to traditional plastics, further enhancing the sustainability of the food supply chain. 

## 2. Seafood Waste-Derived Biopolymers

### 2.1. Chitin

#### 2.1.1. Structure and Sources

**Figure 2:**
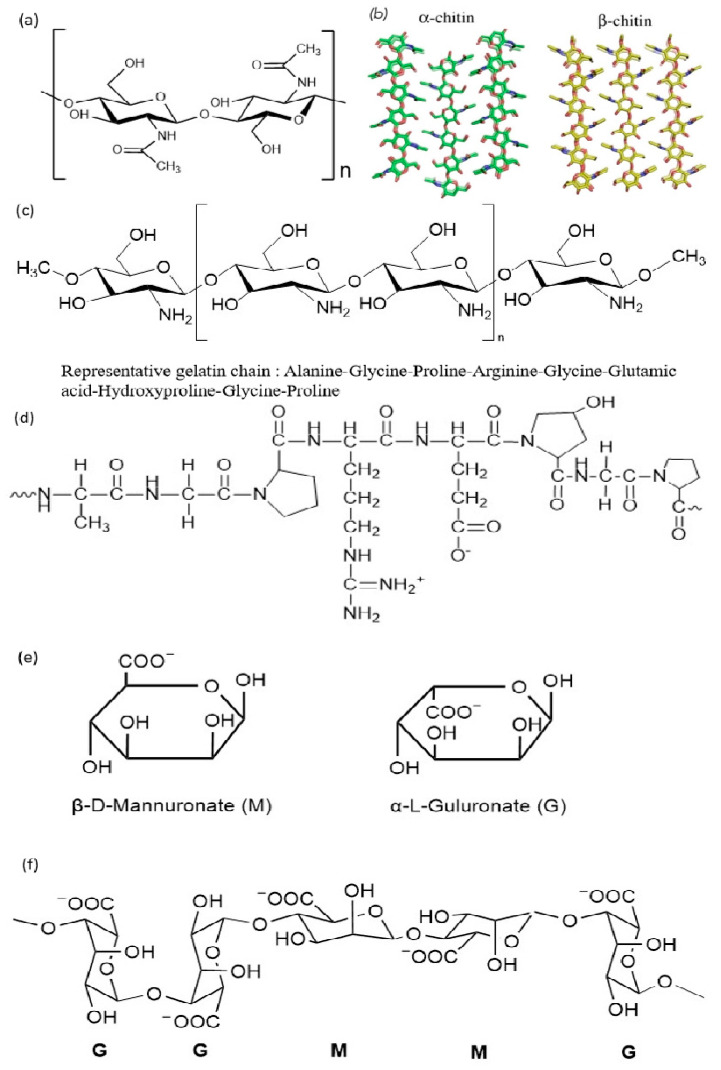
(**a**) The chemical structure of chitin; (**b**) the molecular structure of two common crystal structures of α-chitin and β-chitin [29]; (**c**) the chemical structure of chitosan; (**d**) the representative chemical structure of gelatin; (**e**) the chemical structure of alginate as a single unit; (**f**) the representative alginate chain.

Chitin ranks as the second most abundant natural polysaccharide, following cellulose. It is composed of a linear polysaccharide chain of β-1,4-linked 2-acetamido-2-deoxy-D-glucopyranose units. Abundantly present in nature, chitin exists as ordered crystalline microfibers, serving as strong architectural elements that offer protection and reinforcement in the exoskeletons of arthropods. They can also be found in the casings of mollusks or in the cellular barriers of fungi. The primary function of chitin lies in providing strength and reinforcement to these biological structures. In nature, chitin primarily interacts with proteins to form complex substances. However, depending on the source of chitin, it can also combine with other materials. For example, in crustaceans, chitin combines with calcium carbonate through calcification, thereby reinforcing the hardness of their exoskeletons [24]. Additionally, chitin forms cross-linking complexes with polysaccharides in fungi and binds with polyphenols in insects [25]. As shown in Figure 2, two allomorphs of chitin exist in nature, distinguished by the polarities of the adjacent chains and their packing arrangements. Despite the presence of acetyl groups, chitin remains insoluble in both aqueous and non-polar solutions.

α-chitin stands out as the most abundant form found in nature, distinguished by its widespread occurrence and well-defined structure. This structure consists of closely assembled orthorhombic units, characterized by a pattern of alternating layers of parallel and antiparallel chains that are robustly linked by strong hydrogen bonds within and between the sheets. The presence of these dual types of hydrogen bonding serves to secure the crystal structure along two axes, concurrently inhibiting any expansion when exposed to aqueous environments [26]. α-chitin can be obtained from the exoskeleton of invertebrates like lobsters, crabs, and shrimps, as well as the cell walls of fungi and yeast. α-chitin has been proven to contribute to reinforcing the structure of these exoskeletons [27]. In nature, β-chitin is less commonly found, with squid pens being the most abundant source of this compound [28]. Its structural formation is composed of a monoclinic unit cell, within which the polysaccharide chains are aligned in a parallel fashion.

#### 2.1.2. Extraction Method

The two most common extraction methods for chitin are chemical and biological extraction. The main steps of extraction include demineralization and deproteination. The acid–alkali approach is a commonly employed method [30], favored for its inexpensive reagents and the absence of a requirement for specialized equipment. The raw materials (e.g., crustacean shells) are exposed to high temperatures and acidic conditions for 2–3 h to dissolve and demineralize them. The most common method for deproteination is to use strong bases (e.g., sodium hydroxide) and high temperatures to denature proteins [31]. Chemical extraction is a comparatively low-cost method and very effective in yielding highly purified chitin, which does not require highly specialized equipment [32]. However, the disadvantages of the method include the emission of toxic chemicals, accidental deacetylation of chitin, and probable chain degradation [33]. 

Using ionic liquids to extract chitin is another common chemical method [34]. Numerous ionic liquids have been shown to effectively extract chitin, including 1-ethyl-3-methylimidazolium acetate [35], ethylenediamine [36], alkylimidazolium dimethyl phosphate [37], and 1-allyl-3-methylimidazolium bromide [38]. Ionic liquid methods demonstrate notable advantages, including low vapor pressure, low flammability, and the feasibility of recycling. Even though ionic liquid solvent methods solve the problem of undesired deacetylation, the solvents are toxic to the environment when they are emitted. This method also has a higher cost. In recent years, to address the issues of toxicity and low degradability associated with ionic solutions, deep eutectic solvents (DES) have been proposed as an alternative extraction method [39,40,41,42]. For instance, to reduce acidity and enhance solution degradation and extraction efficiency, a ternary DES composed of choline chloride, glycerol, and either lactic acid or malic acid has been employed for chitin extraction [43].

The biological methods of extraction include the use of either enzymes or fermentation to break down raw materials (e.g., crustacean shells) and separate pure chitin from other components [44,45]. In general, biological methods usually produce chitin with a higher molecular weight and a lower degree of deacetylation than chemical methods [46]. However, enzymatic methods are expensive and cannot completely deproteinate raw materials [47], while fermentation requires a complex medium to achieve optimal purification of chitin and generally takes a long time (usually one week) [44].

#### 2.1.3. Nanochitin

Nanochitin is the general term for chitin whose structure reaches the nanoscale. Nanochitin consists of nanofibers and nanocrystals that form unique nanostructures with morphologies dependent on their source and extraction method [48]. Nanochitin is an emerging nanomaterial distinguished by its intriguing chemical and structural attributes coupled with exceptional applicability. Chemically, the composition of nanochitin enriched with nitrogen and amino groups [49] confers upon its multifaceted processing capabilities and a diverse array of applications. From a structural perspective, nanochitin displays a vast array of dimensions. Its helical and periodic configurations on the surface give rise to a broad spectrum of optical characteristics associated with structural coloring [50,51]. Notably, in the realm of insect physiology, nanochitin facilitates robust adhesion to various surfaces, encompassing both loosely bound and hydrophobic substrates [52,53]. However, nanochitin has its drawbacks, such as high water vapor permeability [54], poor mechanical properties [55], and susceptibility to degradation by water [56]. Nanochitosan demonstrates distinct morphological characteristics, including snowflake-like, rounded, and spherical shapes. Nanochitosan, in comparison to its bulk counterpart (normal chitosan), offers a higher density of reactive functional groups and presents a well-defined nanostructure conducive to interactions with other nanomaterials [57]. While chitin is inherently non-toxic and capable of decomposing, the robust hydrogen bonding among its chains poses a challenge by restricting its solubility. This very characteristic also renders chitin brittle, thereby constraining its range of practical applications [46]. However, nanochitin enhances the tensile strength of biopolymer films and possesses fungicidal and bacteriostatic properties [58]. It also functions as an emulsifying agent, capable of maintaining oil-in-water emulsions’ stability for a duration of up to a month [59].

Nanochitin is produced from fully deacetylated chitin [48]. There are two primary methods for preparing nanochitin: mechanical and chemical. The mechanical approach involves strong shearing to break down raw chitin materials, but this often results in nanochitin with uneven lateral dimensions. Alternatively, the chemical method utilizes controlled, continuous reactions to selectively remove disordered regions of chitin, preserving the crystalline structure [60]. Nanochitin fabrication through acid hydrolysis is a widely employed technique, established by Revol and Marchessault in 1993 [61]. This process produces uniformly distributed nanochitin crystals, which are surface-charged to maintain colloidal stability in aqueous media [62]. Currently, methods such as 2,2,6,6-tetramethylpiperidine-1-oxyl radical (TEMPO)-mediated oxidation [63], the microwave-assisted extraction technique [64], and mechanical disintegration [65] have also been studied and applied to the production of nanochitin.

### 2.2. Chitosan

#### 2.2.1. Structure and Characteristics

Chitosan, the deacetylated form of chitin, which consists of glucosamine and N-acetylglucosamine units [66], as shown in Figure 2, finds widespread application across various industries, most prominently in the food industry [46]. Shrimp and crab are the most common sources of chitosan, but according to existing research, other raw materials such as lobsters and oysters have also been exploited as sources of chitosan. The multifaceted utility of chitosan encompasses various functions critical to several industries. Its efficacy extends to antibacterial and antifungal properties, making it invaluable in food preservation and safety measures. Furthermore, it serves as a versatile food additive with uses as an emulsifier, thickener, stabilizer, color retainer, and natural flavor enhancer. Beyond food applications, it plays a crucial role in water purification, particularly in dye removal processes. Additionally, its integration as dietary fiber contributes to nutritional benefits. Moreover, it could be utilized in the creation of edible films and coverings, providing after-harvest conservation strategies, particularly for fruits [67,68,69,70].

The paramount characteristics of chitosan lie in its degree of deacetylation (DD), measured by the proportion of β-1,4-D-glucosamine present within the polysaccharide chain [71]. Generally, chitin with a DD higher than 50% is considered chitosan [72]. The higher DD indicates higher amino groups in it, demonstrating higher water solubility [73] and stronger biological effects such as antibacterial properties [74].

In addition to the deacetylation degree (DD), the molecular weight (MW) of chitosan constitutes a crucial aspect. Contrary to the trend of DD, chitosan with a smaller MW has stronger biological activity [75]. The water solubility is also related to the MW; the lower the molecular weight, the higher the solubility of chitosan [72]. Upon reaching a molecular weight surpassing 30 kDa, chitosan necessitates the presence of acids, including acetic acid, lactic acid, or citric acid, to facilitate the protonation of its amino groups, thereby enabling water solubility [76].

Chitosan is edible, non-toxic, insoluble in water [77], shows good biocompatibility and biodegradability [76], and has excellent film-forming properties [78], antibacterial activity [79], and antioxidant effects [80].

#### 2.2.2. Extraction Method

Chitosan is produced by the deacetylation of chitin by converting N-acetyl groups into amino groups. Deacetylation techniques can be categorized into both chemical and biological approaches. Conventional chemical treatment involves subjecting chitin to intense alkaline conditions at elevated temperatures, typically surpassing 100 °C [81,82]. Chemical deacetylation generally uses high concentrations of base (up to 70%) [45] for a long time (up to 24 h) [45,82]. The DD corresponds to more aggressive bases, higher temperatures, and longer reaction times [83]. Such rigorous and intense reaction conditions, along with the extended duration, result in a decline in the molecular weight of the produced chitosan [83].

In recent years, microwave radiation extraction technology to extract chitosan has received increasing attention. Microwave radiation technology rapidly heats up the raw materials, and the electromagnetic field causes the materials to vibrate, thereby obtaining deacetylated chitin, i.e., chitosan [84,85]. Recent studies have found that using microwave radiation extraction technology requires lower concentrations of NaOH [86] and can also significantly reduce extraction time [87,88]. Overall, microwave radiation extraction technology could be a greener and more effective way to enhance chitosan extraction.

The biological method of deacetylation is carried out by applying an enzyme, similar to the enzymatic methods of chitin extraction. These enzymes are rare, have low enzymatic activity, and cannot be extracted commercially [89]. Thereby, there is a lot of research focusing on the synergistic use of ionic liquids and enzymatic methods, which could increase the activity of deacetylase and increase the degree of deacetylation of chitosan. Frequently employed ionic liquids include 1-allyl-3-methylimidazolium chloride [90], tetrabutylammonium hydroxide [83], and 1-butyl-3-methylimidazolium acetate [91].

#### 2.2.3. Nanochitosan

Nanochitosan is the general term for chitosan whose structure reaches the nanoscale. The application of chitosan is noteworthy in food packaging, primarily because of its unique composition and beneficial characteristics. These characteristics include its compatibility with living organisms, ability to degrade naturally, and lack of harm to nature [92,93]. Nonetheless, its widespread practical utilization is impeded by inherent limitations such as inadequate mechanical properties, high hygroscopicity, and diminished performance under moisture exposure [94,95]. Addressing these challenges necessitates innovative approaches, and nanotechnology emerges as a promising avenue for overcoming these shortcomings [96,97,98]. Nanochitosan, characterized by reduced particle size, offers improved dispersion within matrices, resulting in enhanced mechanical properties and resistance to deformation [99]. Furthermore, its smaller particle size reduces surface area, mitigating moisture absorption compared to chitosan, thereby enhancing stability and reducing susceptibility to moisture-induced degradation.

Nanochitosan can be prepared by degrading chitosan into low-molecular-weight nanoscale. This can be performed by multiple methods, such as covalent crosslinking, coagulation, precipitation, or ionic cross-linking [100]. Nanochitosan can be extracted by being formed into water-in-oil (W/O) emulsions through cross-linking. Subsequently, a cross-linking agent such as glutaraldehyde, which facilitates droplet hardening, is used to dissolve the nanochitosan [101]. The ionic crosslinking method involves dissolving cationic chitosan in an acidic solvent, followed by its precipitation into particles using an anionic solvent [102]. The initial method was improved by Tian and Groves using sodium sulfate as a precipitation agent to obtain nanochitosan [103]. According to Bodmeier et al., ionic cross-linking nanochitosan is prepared by the cross-linking of the free amino groups of chitosan [104]. The nanoprecipitation method involves dissolving chitosan in a suitable solvent to create a diffusion phase, which is followed by the addition of solvents like polysorbate-80 to produce nanoscale particles [105]. In recent years, advanced technologies such as nano-spray drying have been employed in the production of nanochitosan [106,107].

### 2.3. Gelatin

#### 2.3.1. Structure and Characteristics

Gelatin is a natural polymer composed of amino acids and is a product of collagen that has undergone partial hydrolytic degradation [108] (Figure 2). Gelatin is a compound composed of an amalgamation of individual and dual unfolded peptide sequences, consisting of a spectrum of 18 diverse amino acids. This unique substance exhibits amphipathic properties, as it carries both hydrophilic and hydrophobic groupings alongside both positive and negative electrical charges [109]. 

Gelatin is found in animal scales, connective tissue, bones, intestines, and skin [110], and these parts are often discarded waste products from the seafood industry. Gelatin itself has good film-forming and gelling properties [18,111] and is low in cost, abundant in nature, biodegradable, biocompatible, and non-immunogenic [112]. Previous studies on gelatin-based films discovered that fish gelatin film showed excellent cross-linking capacity, transparency, and UV barrier properties [113]. Gelatin’s unique properties have positioned it as a prospective material for application in the domain of food packaging. Despite its hydrophilic character, gelatin is highly susceptible to moisture, possessing low thermal stability, mechanical resilience, and barrier attributes [114]. Moreover, its brittleness further restricts its usage within the food sector [115].

#### 2.3.2. Extraction Method

Traditionally, the extraction process of gelatin can be divided into two steps: the gelatinization process [116] and the thermal extraction process [117]. The prevalent industrial technique for gelatin extraction is the acid–base methodology. This approach relies on the application of acidic or alkaline agents to break down the covalent bonds within the raw materials, resulting in substantial swelling and solubility of the protein. Then, the gelatin is extracted from the collagen through the process of hot water dissolution [118]. However, the acid–base methodology usually takes a long time (approximately 6–24 h, depending on the source) [119,120]. In addition, the acid–base methodology leads to potential environmental hazards. In recent years, there have been studies working on greener and more efficient ways to extract gelatin, such as using the microwave-rapid freezing–thawing coupling method [121] or by using ultra-high-pressure technology [122].

### 2.4. Alginate

#### 2.4.1. Structure and Properties

Alginate is a natural polymer isolated from the cell walls of brown algae and exists in the form of sodium alginate, calcium alginate, and magnesium alginate. As shown in Figure 2, alginate is a linear, water-soluble anionic polysaccharide composed of α-L-guluronic acid (G) and β-D-mannuronic acid (M) [123]. The U.S. Food and Drug Administration (FDA) has designated alginate as a GRAS (Generally Recognized as Safe) classified substance [124].

The property of alginate to readily gel is attributed to its ability to establish a cross-linking network [125]. Alginate is widely used as a multi-functional ingredient in the food industry. Its applications span various roles, including emulsification, stabilization, thickening, and gelation, thus making it a valuable component in food product formulations [126].

Alginate’s inherent ability to form gels, coupled with its capacity for cross-linking, makes it an ideal choice in the production of biodegradable edible films [125,127,128]. However, due to its poor mechanical properties and hydrophilic nature, pure alginate film has very poor performance [129], especially in regard to its water vapor permeability [130]. Due to this, alginate is mixed with other ingredients in current packaging applications. It is instead added as an antioxidant [131], antibiotic [132], or cross-linking agent [133] to achieve better properties than pure alginate-based films.

#### 2.4.2. Extraction Method

Alginate typically exists in brown algae as calcium alginate but can also be found as sodium alginate, magnesium alginate, and potassium alginate [133]. The primary objective of extraction is to convert insoluble alginic acid into a soluble form, such as sodium alginate. The process typically involves pre-extraction with a weakly acidic solution (e.g., HCl) for 30 min to 24 h, transferring the alginate into insoluble alginic acid, and eliminating non-target compounds such as polyphenols, mannitol, and other readily extractable polysaccharides like laminarin and fucoidan [134]. The subsequent conversion to alginate salts and solution was then followed by the extraction of the alginate from the residue using an alkaline solution such as sodium carbonate, sodium hydroxide, or aluminum hydroxide. Following extraction, alginate is precipitated from the filtrate with the addition of acid, calcium ions, or ethanol [133,135].

## 3. Seafood Waste-Derived Biopolymer Used as Packaging Materials

To serve as food packaging materials, many properties are required. The three most important properties are: (1) mechanical properties; (2) barrier properties of water vapor, gas (oxygen), and oil; and (3) thermal stability. However, due to the inherent characteristics of biopolymers, when they are made into packaging films, they usually have serious deficiencies in these properties that are very important for food films. Therefore, existing research focuses on using various methods to improve the deficiencies in the above properties of films made from biopolymers. The methods of applying these seafood waste-derived biopolymers could be divided into reinforcement materials and base materials. Despite different applications, the targeted properties and mechanisms of improvement remain the same. 

In the realm of packaging materials, there appears to be a lack of research about chitin. This can be attributed to the formidable intermolecular hydrogen bonds inherent in chitin, which greatly constrain its solubility and thus impede its direct applicability as a primary material in film research for packaging purposes. Consequently, researchers typically turn to chitosan as the principal raw material for biopolymer film studies. However, advancements in nanotechnology have spurred interest in nanochitin. Despite its potential, the utilization of nanochitin remains constrained by factors such as cost and processing complexities. As a result, its application is predominantly found as a reinforcing agent within the membranes of alternative biopolymer matrices.

### 3.1. Nanochitin Reinforcement

Because of its special chemical form and biological characteristics [136,137,138], as shown in Table 1, nanochitin can be added as a reinforcement to enhance mechanical properties, oil, water, UV barrier, and antibacterial properties [139]. There have been many previous studies on the combination of nanochitin and artificial polymers into nanocomposite films to enhance performance, such as adding polylactic acid [140] and polyvinyl alcohol [139], to improve films’ barrier properties and antibacterial properties.

In the context of food packaging, the primary objective revolves around safeguarding against and prolonging the deterioration of enclosed edibles. Consequently, the crucial attributes of biopolymer films lie in their oxygen and water vapor transmission rates, as these factors directly dictate the rate of food spoilage within the packaging [154]. With the addition of nanochitin, most studies aim to improve barrier properties by reducing the penetration of oxygen and water vapor. This is achieved by increasing cross-linking between polymer chains [155] or introducing a tortuous path for water and oxygen molecules [156].

Many studies have applied this theory and shown great improvement in reducing water vapor permeability. With the escalation of nanochitin content from 0% to 2%, Qing et al. observed a decline in water vapor permeability from 5.32 × 10^−12^ to 2.22 × 10^−12^ g m^−1^ s^−1^ Pa^−1^ [149]. With the incorporation of 5% of nanochitin, Oun and Rhim reported that the water vapor permeability of carboxymethyl cellulose decreased from 1.92 × 10^−9^ to 1.47 × 10^−9^ g m^−1^ Pa^−1^ s^−1^ [142]. According to Bahrami et al., the integration of nanochitin had the ability to mitigate the hygroscopic characteristics of starch. They also observed a significant decrease in moisture absorption, from 51% down to 38%, upon the addition of 5% nanochitin. This improvement was attributed to enhanced hydrogen bonding and the subsequent reduction in the free space between starch molecules [151]. Sahraee et al. incorporated corn oil to overcome the hydrophilic nature of gelatin and nanochitin and to reduce its propensity to water molecules, resulting in a decrease in water vapor permeability from 8.89 × 10^−10^ g s^−1^ m^−1^ Pa^−1^ to 7.68 × 10^−10^ g s^−1^ m^−1^ Pa^−1^ [146]. Jiang et al. proved that the carboxyl group of citric acid condensed with the amino group in nanochitin forms an amide bond that creates a structure that is more uniform and denser. From this discovery, water vapor permeability was reduced from 6.04 × 10^−11^ g m^−1^ s^−1^ Pa^−1^ to 3.48 × 10^−11^ g m^−1^ s^−1^ Pa^−1^ [148].

Nanochitin exhibits remarkable stiffness and tenacity, primarily due to its high degree of crystallinity and robust intramolecular hydrogen bonding network [23,58]. Therefore, adding a certain concentration of nanochitin to the film can effectively enhance its mechanical performance, boosting its tensile strength and elongation at the break [137,139,141]. Hai et al. revealed a positive correlation between nanochitin concentration and both Young’s modulus and tensile strength, demonstrating an increase in these properties with an increase in the nanochitin content [141]. Etxabide et al. claimed that with the addition of nanochitin, gelatin film can achieve similar mechanical properties to those of conventional plastic [113]. In the Bahrami et al. study, with the addition of 5% nanochitin, Young’s modulus of the composite film increased by 239%, and the tensile strength increased by 216% [151]. The investigation by Xin et al. revealed that the stress transfer was significantly enhanced with evenly distributed nanochitin, resulting in a 50.3% augmentation in tensile strength and a remarkable 174% boost in elongation at break when contrasted with films lacking nanochitin [153]. However, because the nanofibers are difficult to distribute evenly and are easy to agglomerate at high concentrations, adding a high concentration of nanochitin could reduce the mechanical properties of raw composite materials [58,98,111,157].

In conclusion, the incorporation of nanochitin has significantly enhanced the various properties of biodegradable packaging films. Most studies utilized casting and drying methods for film fabrication. The observed improvement in mechanical properties might be due to increased cross-linking between nanochitin and biopolymers, as nanochitin fills the structural gaps within biopolymers [137,139,141]. Barrier performance is also enhanced by nanochitin filling the structural gaps within biopolymers and creating a tortuous pathway [151]. As shown in Table 1, notable enhancements with nanochitin reinforcement include improved mechanical strength, barrier properties, thermal stability, solubility, and moisture absorption capacity. Furthermore, as shown in Table 1, the addition of nanochitin imparts antioxidant [143,153,158], antibacterial [113,144,146,147,148,149,152], thermal stability [141,142,145,147,158], and UV-blocking properties [113,142,143,145] to the film, which enhances its effectiveness in food packaging applications, leading to an extended freshness duration for delicate edibles.

### 3.2. Chitosan-Based Packaging

As a relatively well-established alternative packaging material, extensive research has already highlighted chitosan’s biocompatibility, biodegradability [76], excellent film-forming properties [78], antibacterial activity [79], and antioxidant effects [80]. Despite its numerous advantages, chitosan utilization in food packaging faces inherent limitations, notably its pronounced susceptibility to moisture and its inferior mechanical and thermal stability [159,160]. These drawbacks impede its wider adoption in packaging applications, necessitating further research to address these challenges and unlock its full potential in enhancing food safety and preservation. Some strategies include cross-linked chitosan [161,162,163], graft copolymerization [164,165], enzyme treatment [166], and blending with biopolymers [167,168,169,170,171,172,173] (Table 2).

The cross-linked film composed of chitosan and polysaccharide primarily relies on intermolecular interactions to form a cohesive polymer network. Chitosan features numerous amino and hydroxyl groups along its molecular chain, facilitating the formation of amide and hydrogen bonds with various molecules. It also undergoes etherification and esterification reactions, resulting in covalent bond formation. This network is predominantly strengthened by intermolecular electrostatic interactions. The synergistic effect arising from these interactions leads to enhanced film thermal stability and mechanical properties [168,174]. The integration of eugenol and ginger oil enhances the tensile strength of the film while reducing its stiffness in comparison to films solely composed of chitosan [168]. The findings of Younis and Zhao revealed a prevalent occurrence of synergistic enhancements in the mechanical performance attributes of edible films through the incorporation of chitosan and pectin [173]. The results of Zimet et al., Wu et al., and W. Li et al. also proved that the interactions caused by the addition of other biopolymers notably improve the mechanical properties of chitosan-based films [177,178,179]. These intermolecular interactions and the potential of chitosan could form more intermolecular interactions, giving chitosan-based films great potential to improve their mechanical and other properties.

Numerous studies have shown that augmenting chitosan-based films with additional ingredients enhances mechanical properties and thermal stability, even providing the film with additional valuable properties including antibacterial abilities [166,171,175,177,178], antioxidant effects [166,179], better water vapor permeability [171,173,175,178,179], and a stronger UV barrier [168]. Through synergistic effects and interactions, such approaches hold promise for addressing stiffness limitations and achieving mechanical properties comparable to petroleum-based plastics in future chitosan-based films.

### 3.3. Chitosan Reinforcement

Chitosan exhibits multifaceted potential in film matrices due to its strong connective properties and inherent antibacterial attributes. Incorporating chitosan into biopolymer-based films shows promise for enhancing overall performance. Recent research has emphasized its synergistic effects when combined with protein-based films, underscoring avenues for further exploration and optimization. The prominence of proteins in the creation of degradable films is noteworthy, primarily due to their innate characteristics such as commendable mechanical strength and superior barrier properties against oxygen, carbon dioxide, and volatile scent molecules. In particular, chitosan–protein blend films exhibit enhanced functional characteristics compared to their individual protein or chitosan films. Table 3 encapsulates an exhaustive examination of the characteristics and effectiveness exhibited by chitosan–protein composite films.

Existing studies have proven that chitosan can form intermolecular hydrogen bonds and other links with proteins, thereby improving the performance of the film. An intermolecular hydrogen bond occurs between the ammonium groups in the chitosan backbone and the hydroxyl groups present in proteins like gelatin and peptides [188]. The research conducted by Batista et al. established that protein and chitosan concentrations substantially contribute to the enhancement of the film’s mechanical attributes. Their findings indicated an increase in chitosan levels, which correspondingly led to higher tensile strength measurements [182]. Furthermore, the carboxylate moieties in chitosan engage in electrostatic interactions with the amino units of the protein, thereby giving rise to a polyionic complex. This process of interconnection results in a diminution of the available free space within the film’s architecture, ultimately enhancing the density of the structural framework. As a result, the increased density within the film significantly hampers the diffusion pace of water molecules [180,183,184,185] and also contributes to an increase in tensile strength [182,183,184].

While chitosan alone has limitations in film formation, blending it with other biopolymers enhances its properties and overcomes some of its limitations. This combination leverages the strengths of each polymer, resulting in composite films with superior performance compared to individual components. Simple blending and casting methods can achieve molecular cross-linking and synergistic effects between components. However, the casting and drying method requires 48–72 h to fully dry at room temperature, posing a challenge for large-scale production. 

### 3.4. Gelatin-Based Film

Gelatin exhibits robust gas barrier characteristics and superior film-forming abilities. Despite its advantages, gelatin encounters shortcomings in terms of mechanical resilience and hygroscopicity [109], thus constraining its application as a packaging substance. Previous research efforts have primarily focused on utilizing proteins, polysaccharides, carbohydrates, and phenolic compounds to improve both the physical properties and biological characteristics of gelatin-based film systems (Table 4).

Table 4 emphasizes the key improvement domains in gelatin-based films, particularly focusing on reinforcing their feeble mechanical strength and enhancing their insufficient water vapor permeability [109]. Due to gelatin’s abundance of connectable bonds, strategies involving the cross-linking of hydrogen and hydrophobic bonds have been explored. Incorporating additional materials into the gelatin network effectively mitigates its inherent brittleness and enhances its mechanical properties [113,144,183,189,191,194]. Nisin A^®^ was integrated into the design of gelatin films by Etxabide et al., which resulted in a decrease in gelatin’s crystalline nature and the induction of a more disordered, amorphous structure. This integration resulted in increased elongation at break, observed to rise from 44.50 ± 7.30 to 112.26 ± 5.47. Furthermore, the integration of Nisin A^®^ resulted in decreased measurements for both tensile strength and elastic modulus, suggesting an increased suppleness in the gelatin film [113]. Sara Khedri et al. revealed that the interplay of phosphate components from bioactive casein phosphopeptides (CPPs) with gelatin’s functional groups, involving both covalent bonds and hydrogen bonds, significantly strengthens the structural stability of the composite substance. The incorporation of bioactive CPPs at concentrations below 0.2% contributes to the enhancement of mechanical properties, with optimal improvements in elongation at break (359.8%) and tensile strength (188.54%) achieved at a 0.1% concentration [189].

Furthermore, the introduction of nanomaterials into the gelatin matrix serves to augment water vapor permeability by rendering the pathway more tortuous [144,183]. Azarifar et al. identified that the film composition consisting of 10% carboxymethyl cellulose, 6% chitosan nanofiber, and 0.63% α-juniper essential oil (AJEO) demonstrated the minimum water vapor permeability value, recorded at 0.67 × 10^−7^ g/(mhPa). However, beyond this AJEO concentration, an increase in oil content led to a rise in WVP due to the formation of voids at the interface of the hydrophilic–hydrophobic surface, thereby diminishing barrier properties against water vapor transmission [144]. Haghighi et al. observed that the water vapor permeability of chitosan–gelatin and chitosan–gelatin/lauroyl arginate ethyl films decreased by 22.47% and 25.08%, respectively, compared to pure gelatin films. Apart from the mechanisms previously discussed, potential contributions from hydrogen bonding and covalent interactions were noted, further attenuating the accessibility of hydrophilic groups [183].

As shown in Table 4, the incorporation of biopolymers with gelatin-based film could improve the properties and showed great potential as a food packaging candidate. Significantly, the integration of specialized ingredients like essential oils and nanoparticles endows gelatin-based films with distinct features, namely antioxidant capabilities and antibacterial attributes [143,191,192,195], rendering them more suitable for food packaging applications. The methods used in these studies typically involve mixing, casting, and then drying, with the drying process at room temperature taking no less than 48 h. However, gelatin’s excellent gelling properties enable rapid film formation, which can also present challenges for ingredient mixing and large-scale production. For instance, the high viscosity of gelatin requires careful temperature maintenance during mixing and controlled casting time to ensure completion before the temperature drops. These factors complicate the scalability of production processes.

### 3.5. Alginate-Based Film

Alginate exhibits several properties that make it highly suitable for film production. These include excellent film-forming capabilities, notable tensile strength, flexibility, and biocompatibility, as well as being odorless and tasteless and possessing commendable biodegradability [196]. However, a significant drawback of alginate-based packaging lies in its elevated water vapor transmission rate, stemming from its inherent hydrophilicity. This attribute renders alginate susceptible to water absorption and swelling, particularly in high-humidity environments, thus compromising its efficacy in food packaging applications [130]. Moreover, packaging derived solely from alginate often presents challenges associated with strong mechanical and barrier properties, along with limited antimicrobial efficacy [197]. To address these shortcomings, various effective enhancements have been proposed and implemented (Table 5).

Numerous studies have investigated the modification of alginate film through the addition of various substances to induce distinct cross-linking mechanisms. These mechanisms include hydrogen bonding [198,199,207], physical cross-linking [125,201,204], and the incorporation of specific components, such as thymol, to influence crystal structure [206]. Furthermore, research has explored strategies such as gap filling within alginate matrices to enhance mechanical properties [125,198,206], thermal stability [125,203], water vapor permeability [125,206,207], and oxygen barrier properties [203]. Additionally, these modifications have demonstrated functionalities including UV barrier [125,205,206], antioxidant properties [206], and antibacterial activities [204,207]. Enhancing the performance of alginate-based films involves increasing cross-linking, such as hydrogen bonding, to improve mechanical strength and barrier properties. Alginate’s solubility varies with different cation combinations; for instance, calcium alginate is completely insoluble. Utilizing this property, Dai et al. replaced Ca^2+^ ions with Na^+^ ions in sodium alginate, forming an insoluble hydrogel and reinforcing it with hydrogen bonds [199]. This characteristic allows alginate to create a more tortuous path within the film, thereby enhancing its barrier performance and having similar effects and mechanisms as the addition of nanomaterials. As a result, the composite film demonstrates increased stability and improved resistance to water permeation. 

The unique properties of alginate, especially its variable solubility with different cations, could play a more crucial role in forming more effective barrier films. By strategically manipulating these properties and corresponding with design desire bonding, researchers can develop films with superior performance characteristics and achieve the same effect and mechanism without using nanomaterials, making alginate-based materials highly versatile and effective for various applications.

Notably, Tong et al. established that their film samples share comparable characteristics with polyethylene plastic films in terms of their thickness, hue, moisture content, solubility, water vapor transmission rate, bending strength, and tensile elongation. The integration of pectin and alginate with sugars that encompass aldehyde groups promotes intramolecular connections, resulting in the creation of hydrogels through their interactive processes. Notably, the compatibility and chemical synergy observed between these components and cinnamic acid contribute to enhanced mechanical properties. Moreover, the inclusion of cinnamic acid enhances the hydrophobic nature of the films, thereby effectively increasing their ability to resist water vapor transmission [204].

### 3.6. Antimicrobial Properties of Bio-Based Film

For food packaging, one of its primary functions is to prevent spoilage. Incorporating antibacterial agents into the packaging materials significantly enhances their protective capabilities. Many biopolymers and ingredients, such as nanochitin, chitosan, and nanochitosan, have antimicrobial properties [178], making the derived films desirable for food packaging. On the other hand, some biopolymers, such as gelatin, do not inherently possess antimicrobial activities; the antimicrobial properties of these biopolymer-derived films usually come from added ingredients like pomegranate peel extract [181], lauroyl arginate ethyl [183], essential oil, and nano metal ions. Most antibacterial testing of films focused on the food-borne pathogens *L. monocytogenes* and *E. coli* [141,142,148,149], as well as the fungus *A. niger* [146,158]. 

The mechanisms behind the antimicrobial properties are quite different. For biopolymers such as nanochitin, it has positive charges, and interactions can be formed between the positive charges of nanochitin and the negative charges of bacterial cell membranes, leading to the rupture of bacterial cells and leakage of intracellular material, contributing to the death of bacteria. While chitosan is derived from the deacetylation of chitin, chitosan possesses a unique polycationic nature that is absent in chitin [208]. This distinct characteristic accounts for their significantly different antibacterial mechanisms. Chitosan and nanochitosan could promote the separation of microbial cell walls and bind with the proteins inside the microbe, compromising the cell membrane’s ability to regulate the passage of substances, ultimately resulting in bacterial cell death [198]. In addition to having a direct effect on bacteria, adhesion to bacteria also provides antibacterial activity. Hydrophilic surfaces and the introduction of hydrophilic carboxymethyl groups might also contribute to the improvement of its antimicrobial activity, such as against *L. monocytogenes*, owing to its peptide’s tendency to adhere to hydrophobic surfaces [177]. 

In addition to utilizing the biopolymer itself for antibacterial properties, various other additives are incorporated to enhance antibacterial effects. Ahmad et al. revealed zinc oxide nanoparticles release Zn^2+^ and bind with the negatively charged bacterial cell wall, leading to the disruption of the microbes, which gives the film antimicrobial properties [158]. The addition of peanut red skin extract enhanced the membrane permeability and led to the fragmentation of DNA as well as the oxidation of lipids [199]. To summarize, different polymers may have diverse antimicrobial mechanisms. By incorporating these biopolymers into packaging development, we can prevent food spoilage and enable longer-distance transportation.

## 4. Limitations and Future Directions

Despite their promising attributes, biopolymer films face inherent limitations that hinder their widespread adoption as substitutes for plastics. These challenges encompass the hydrophilic nature and weak mechanical properties of biopolymers, scalability issues in manufacturing processes, and constraints in comprehensive property comparisons with plastics, and thus they still fall short in replicating the barrier and mechanical characteristics offered by conventional plastics.

However, there are many current studies that have achieved significantly better results in improving films. A study by Azarifar et al. demonstrated that gelatin-based films incorporating nanochitin can achieve water vapor permeability comparable to, or even surpassing, that of conventional plastic films like Polyethylene Terephthalate (PET), which typically has a permeability of around 18 g/(m²·day) [144]. Additionally, Tong et al.’s research on alginate-based films indicated that their solubility, water vapor permeability, flexural strength, and elongation at break are equivalent to those of polyethylene films. These findings highlighted the potential of biopolymer films to meet or exceed the performance of traditional plastic materials [204]. Through the research of adding different ingredients, some interesting properties of the films, such as antioxidant abilities, UV resistance, and antibacterial abilities, have been obtained.

Currently, the enhancement of bio-based films is primarily achieved by incorporating functional ingredients that facilitate various forms of cross-linking, such as covalent and hydrogen bonds. In addition, the incorporation of nanomaterials such as nanochitin and nanochitosan enhances barrier properties by creating tortuous paths inside the films. However, the improvement is not significant. On the other hand, there is limited research on film fabrication methods, with nearly 90% of current research focusing on simple blending followed by casting. Applying alternative processing methods such as layer-by-layer assembly [209], electrospinning [210], and solvent evaporation [211] to filmmaking may further uncover the potential of biopolymers. Finally, layer-by-layer assembly with bio-based plastics like poly lactic acid [212], poly trimethylene terephthalate [213], and poly hydroxybutyrate [214] could have the potential to further improve performance.

The food packaging materials discussed in this article are prefabricated films, which allow for direct use in food packaging during the production process. Despite advancements in bio-based films, thermal stability remains a challenge. For instance, in many bio-based films, the first stage of decomposition usually starts at 40–70 °C and fully decomposes between 400 °C and 600 °C [158,182,185]. The glass transition temperature of gelatin-based films is approximately 120 °C [215]. Typically, there will be sterilization/pasteurization processes for raw materials before or after packaging, which can expose materials to temperatures around 100 °C. Therefore, selecting bio-based packaging requires careful consideration of thermal stability and the implementation of cooling measurements during processing to maintain material integrity. Future research can explore the use of unique raw materials as well as strengthening the cross-linking between materials in bio-based films.

The existing laboratory research on these biopolymer films derived from seafood waste has not yet transitioned to large-scale production due to several factors. Firstly, the lack of a film with exceptional performances across all aspects suitable for promotion impedes progress. Overcoming the inherent hydrophilic properties and the poor mechanical properties (e.g., stiffness and inherent brittleness) of biopolymer films remains a significant challenge at the same time. Secondly, the current method primarily involves simple blending and casting, followed by prolonged drying at room temperature for 48–72 h, rendering it inefficient for large-scale production. The molding of biopolymer films is constrained by specific drying conditions, further hindering large-scale industrial production. Furthermore, innovative film-making methods such as layer-by-layer assembly, solution blow spinning, thermal evaporation, and spin coating face significant challenges for large-scale industrial production. For instance, layer-by-layer assembly is time-intensive [216], while thermal evaporation demands high energy consumption and the complexity of maintaining a vacuum environment [217,218,219]. Nonetheless, a key point of future research is the need to focus on how to further expand these laboratory research results and put them into industrial production. For instance, optimizing and simplifying critical steps (e.g., vacuum environment, time consumption, etc.) that currently restrict industrial production may be a huge step forward for their use in large-scale production.

The production cost of bio-based packaging materials is a critical factor to consider as well. The unit cost of plastic in its primary form is just over $1000 per ton [220]. Given the low cost and wide availability of petroleum-based plastics, any alternatives must be economically competitive. Therefore, the cost of bio-based packaging should be comparable to or less than that of traditional petroleum-based plastics to be viable for widespread adoption. The literature on biopolymer production often overlooks the associated costs, particularly in the context of food packaging films. There are few detailed discussions on production costs, and existing research is primarily at the laboratory scale. It is important to acknowledge that the costs of large-scale production may differ significantly from those observed in laboratory settings, which leads to a lack of theoretical cost projections for biopolymer film large-scale operations.

## 5. Conclusions

Utilizing seafood waste biopolymers is a promising way to develop substitute packaging materials for petroleum-derived synthetic plastics. Here, we have reviewed the structure, characteristics, extraction method, and recent application advances in packaging. These biopolymers possess intriguing properties such as degradability, antimicrobial activity, and chelating capabilities. Moreover, their film-forming properties and robust mechanical strength render them suitable for application in food packaging materials.

This article categorizes films made from various biopolymers and reviews existing research focused on the enhancement of their properties. We note that most property improvements are achieved by forming a bond between the biopolymers and/or filling molecular gaps to create more tortuous paths. Incorporating these biopolymers as reinforcement or as base materials results in additional bonding, such as hydrogen and covalent bonding, creating a more uniform and denser structure. This increased bonding enhances barrier properties, tensile strength, and elongation at break, as well as the potential possibility of structural stability and thermal stability improvement. However, the material stiffness (e.g., Young’s modulus) also increased with more bonding. Some of these biopolymer films could achieve the same properties as petroleum-derived plastics in some areas, such as water vapor transmission rate, bending strength, and tensile elongation.

We anticipate that this biopolymer film derived from seafood waste offers a sustainable packaging solution, adding value to seafood processing while mitigating environmental impact. Despite their potential, biopolymer films face significant challenges in large-scale adoption due to their inherent hydrophilic nature, weak mechanical properties (e.g., stiffness and inherent brittleness), weak stability (structure and thermal), inefficient production processes, and higher production costs compared to conventional plastics. Addressing these challenges, especially scalability and cost-effectiveness, is essential for their broader application. Future research should also focus on techno-economic analysis and resolving technical limitations associated with scaling up production. We hope our review will inspire researchers in this area and advance the development and commercialization of seafood waste-derived food packaging.

## Figures and Tables

**Table 1:** Summary of studies on improving the properties of nanochitin-reinforced biopolymer-based films.

Ingredients	Properties Improvement	Reference
Bamboo cellulose nanofiber	**Mechanical properties:** tensile strength increased by 204%, Young’s modulus increased by 33%, elongation at break increased by 152%; **Biodegradable time:** fully degraded within a week; **Antimicrobial properties:** *Escherichia coli* (*E. coli*) and *Listeria monocytogenes* (*L. monocytogenes*).	[141]
Carboxymethyl cellulose, ZnO-Ag	**Mechanical properties:** tensile strength increased by 132%, elastic modulus increased by 101%; **Water vapor permeability:** decreased by 21%; **UV light barrier**; **Thermal stability:** mid-point temperature of the degradation increased by 15 °C.	[142]
Gelatin	**Mechanical properties:** elongation at break increased by 152%, elastic modulus increased by 140%; **UV light barrier**.	[113]
Gelatin, anthocyanins	**Mechanical property:** elongation at break increased by 23%; **UV light barrier:** light transmittance rate at 560 nm decreased by 60.18%; **Oxygen barrier:** decreased by 35%; **Water vapor permeability:** decreased by 14%; **Antioxidant property:** the maximum radical scavenging rate could be close to 100%.	[143]
Gelatin, carboxymethyl cellulose, ajowan essential oil	**Mechanical properties:** ultimate tensile strength significantly decreased, and strain at break increased; **Water vapor barrier:** water contact angle increased and water vapor permeability decreased by 40%; **Antibacterial properties:** *Staphylococcus aureus* (*S. aureus*) and *E. coli*.	[144]
Glucose-crosslinked gelatin	**Thermal stability:** degradation temperature increased; **UV light barrier**; **Mechanical properties:** Young’s modulus increased by 98%, ultimate tensile strength increased by 83%; **Antioxidant release:** absorbance in the UV region increased and reduced the occurrence of photo-oxidation reactions in foods.	[145]
Gelatin, corn oil	**Mechanical properties:** elastic modulus decreased by 82% and elongation at break increased by 887%; **Moisture content:** decreased by 51%; **Water vapor barrier:** solubility and water contact angle increased by 33%, and water vapor permeability decreased by 21%; **Antimicrobial property:** *Aspergillus niger*.	[146]
Gelatin, zinc oxide nanoparticles	**Mechanical properties:** ultimate tensile strength increased by 2%, elongation at the break increased by 194%, elastic modulus increased by 175%; **Water vapor permeability:** decreased by 44%; **Thermal stability:** temperature of melting point increased by 25% and enthalpy increased by 1150%; **Antimicrobial property:** *A. niger*.	[147]
Konjac glucomannan, citric acid	**Mechanical properties:** tensile strength increased by 191.86%, elongation at break increased by 444%; **Water vapor permeability:** 42% reduction; **Antibacterial properties:** *S. aureus* and *E. coli.*	[148]
Maize starch	**Mechanical property:** tensile strength increased by 45%; **Water vapor permeability:** decreased by 58%; **Antibacterial properties:** *L. monocytogenes* and *E. coli*.	[149]
Nanocellulose, nano-SiO_2_	**Mechanical property:** tensile strength increased by 80%; **Light transmittance:** showed 30.7% lower; **Super hydrophobicity:** the water contact angle becomes larger, increasing by 106.4°.	[150]
Starch	**Mechanical properties:** tensile strength increased by 216%, toughness had a 270% reduction, Young’s modulus increased by 239%; **Moisture absorption:** moisture absorption dropped 13%.	[151]
Zein	**Antibacterial properties.**	[152]
Zein, potato starch	**Mechanical properties:** tensile strength increased by 66%, elongation at break increased by 163%; **Barrier properties:** water vapor permeability decreased by 10%, oxygen permeability decreased by 17%; **Antioxidant characteristic.**	[153]

Improvement percentages are calculated by comparing the best performance for each property to the control sample.

**Table 2:** Summary of studies on improving the properties of chitosan-based film through cross-linking and blending with other ingredients.

Ingredients	Properties Improvement	Reference
Acetic acid, glycerin	**Antibacterial properties:** *Pseudomonas aeruginosa* (*P. aeruginosa*), *Pantoea ananatis*, and *E. coli*; **Antioxidant property:** DPPH reduction barely exceeds 10%.	[166]
Carboxymethyl cellulose, glutaraldehyde, cinnamon essential oil, oleic acid	**Mechanical property:** elongation at break increased by 62%; **Solubility:** 32% reduction; **Antibacterial properties:** *P. aeruginosa* and *L. monocytogenes*; **Antioxidant property.**	[171]
Carvacrol and xylan	**Mechanical properties:** tensile strength increased by 41%, elongation at break increased by 14%; **Thermal stability:** mass loss rate decreased by 14.5%/°C.	[174]
Eugenol, ginger essential oils, gelatin	**Mechanical properties:** tensile strength increased by 106%, elongation at break increased by 822%; **UV barrier:** transmittance was null until 300 nm and showed a high barrier from 300–450 nm.	[168]
Gum, cinnamon, and clove essential oils	**Mechanical property:** elongation at break increased by 28%; **Antibacterial properties:** *S. aureus* and *E. coli*; **Water vapor permeability:** 36% reduction.	[175]
Gelatin, tapioca starch, zinc oxide nanoparticles	**Mechanical properties:** tensile strength increased by 10%; **Thermal stability:** melting temperature increased by 4%; **Antibacterial properties:** *S. aureus* and *E. coli.*	[158]
High-methoxyl apple pectin	**Mechanical properties:** tensile strength increased by 432%, elongation at break increased by 62%, Young’s modulus increased by 358%; **Water barrier:** water vapor permeability decreased, and water contact angle increased; **Transparency:** opacity increased by 345%.	[173]
Hordein, quercetin	**Water resistance:** water contact angle increased; **Antioxidant activity:** Slowing down the enzymatic browning rate of food.	[176]
Mango leaf extract	**Mechanical properties:** tensile strength increased by 27%, elastic modulus increased by 23%; **Water barrier:** water vapor permeability decreased by 52%, water contact angle increased by 17.1°; **Antioxidant activity:** opacity increased by 78%.	[93]
Nisin	**Mechanical properties:** tensile strength increased by 23%, elongation at break increased by 211%; **Thermal stability:** endotherm peak temperatures increased by 8.8 °C; **Antibacterial property:** *L. monocytogenes*.	[177]
Potato starch, citric acid	**Mechanical properties:** tensile strength increased by 7%, elongation at break increased by 57%; **Water barrier:** water contact angle increased by 17.26°, water vapor permeability decreased by 30%; **Antimicrobial properties:** *E. coli* and *S. aureus*.	[178]
Starch, nano titanium dioxide, clove oil	**Mechanical properties:** tensile strength increased by 19%, elongation at break increased by 45%; **Water barrier:** water contact angle increased by 23.5°, water vapor permeability decreased by 25%; **Antioxidant activity:** DPPH radical scavenging activity increased by 222%, the ABTS radical scavenging activity increased by 130%.	[179]

Improvement percentages are calculated by comparing the best performance for each property to the control sample.

**Table 3:** Summary of studies on improving film properties through adding chitosan to protein-based films.

Ingredients	Properties Improvement	Reference
Eggshell membrane gelatin	**Mechanical property:** elongation at break increased by 1031%; **Water vapor permeability:** 84% reduction; **UV barrier.**	[180]
Fish collagen, pomegranate peel extract	**Antibacterial properties:** *Bacillus saprophyticus* LNB 333 F5, *Bacillus subtilis* NCIM 2635, *Salmonella typhimurium* (*S. typhimurium*) NCIM 2501, *E. coli* NCIM 2832.	[181]
Fish residue myofibrillar proteins	**Mechanical properties:** tensile strength increased by 28%, elongation at break increased by 179%; **Solubility:** 66% reduction; **UV barrier:** except for the wavelength at 280 nm, all transmittance values were significantly lower; **Thermal stability:** the melting temperature was higher.	[182]
Gelatin, lauroyl arginate ethyl	**Mechanical properties:** tensile strength increased by 258%, elongation at break increased by 9%, elastic modulus increased by 72%; **Water vapor permeability:** 25% reduction; **UV barrier**; **Antibacterial properties:** *L. monocytogenes*, *E. coli*, *S. typhimurium*, *Campylobacter jejuni* (*C. jejuni*).	[183]
Polyphenols	**Mechanical properties:** tensile strength increased by 21%, elongation at break increased by 171%; **Water vapor permeability:** 54% reduction; **Antioxidant activity**: DPPH radical scavenging activity increased by 2070%.	[184]
Porcine plasma protein	**Mechanical properties:** tensile strength increased by 651%, elongation at break increased by 163%; **Water solubility**; **Water vapor permeability:** 50% reduction; **Thermal ability:** 1st peak temperature and 2nd peak temperature increased by 3 °C.	[185]
Whey protein	**Mechanical property:** tensile strength increased by 141%; **Antioxidant activity:** DPPH activity increased by 113%.	[186]
Zein, α-tocopherol	**Oxygen barrier:** oxygen permeability decreased by 61%; **Water vapor permeability**: 50% reduction **Antioxidant activity:** inhibit the browning of mushrooms, which may be related to the inhibition of POD and PPO activities.	[187]

Improvement percentages are calculated by comparing the best performance for each property to the control sample.

**Table 4:** Summary of studies on improving the film properties of gelatin-based films.

Ingredients	Properties Improvement	Reference
Anthocyanins, nanochitin	**UV light barrier:** light transmittance rate at 560 nm decreased by 60.18%; **Oxygen barrier:** decreased by 35%; **Water vapor permeability:** 14% reduction; **Antioxidant property:** the maximum radical scavenging rate could be close to 100%.	[143]
Casein phosphopeptides	**Mechanical properties:** tensile strength increased by 89%, elongation at break increased by 260%; **Water vapor permeability:** 35% reduction; **UV barrier**; **Antimicrobial properties:** *Bacillus cereus* (*B. cereus*) and *S. aureus*; **Antioxidant activity.**	[189]
Carboxymethyl cellulose, nanochitin, ajowan essential oil	**Mechanical properties:** ultimate tensile strength significantly decreased, and strain at break increased; **Water vapor barrier:** water contact angle increased, and water vapor permeability decreased by 40%; **Antibacterial properties:** *S. aureus* and *E. coli*.	[144]
Chitosan, citric acid	**Swelling:** swelling values decreased below 600% after 24 h with the addition of citric acid; **UV Barrier:** films provided a UV light barrier from 200 to 250 nm; **Antibacterial property:** *E. coli*; **Hydrophobic surfaces.**	[190]
Chitosan, gallic acid	**Mechanical property:** Young’s modulus increased by 103%; **Water vapor permeability:** 12% reduction; **Antioxidant property**; **Antimicrobial properties:** *B. cereus*, *S. aureus*, *E. coli*, and *S. typhimurium*.	[191]
Chitosan, nisin, frape seed extract	**Antioxidant activity.**	[192]
Chitosan, lauroyl arginate ethyl	**Mechanical properties:** tensile strength increased by 258%, elongation at break increased by 9%, elastic modulus increased by 72%; **Water vapor permeability:** 25% reduction; **UV barrier**; **Antibacterial properties:** *L. monocytogenes*, *E. coli*, *S. typhimurium*, *C. jejuni.*	[183]
Corn starch, guabiroba pulp	**Mechanical property:** tensile strength achieved highest at gelatin: corn starch = 2:1, elongation at break reached highest at gelatin: corn starch = 1:2; **Water vapor permeability:** achieved lowest at gelatin: corn starch = 1:1.	[193]
Di-aldehyde nanocellulose	**Mechanical Properties:** tensile strength enhanced 275%; **Hydrophilic property**.	[194]
Nanochitin	**Mechanical properties:** elongation at break increased by 152%, elastic modulus increased by 140%; **UV light barrier**.	[113]
Titanium dioxide doped silver nanoparticles	**Thermal stability:** glass transition temperature increased by 82 °C, film is thermally stable until approximately 800 °C; **Antioxidant property:** DPPH radical scavenging activity increased by 25%.	[195]
Tapioca starch, nanochitin	**Mechanical property:** tensile strength increased by 10%; **Thermal stability:** melting temperature increased by 4%; **Antibacterial properties:** *S. aureus* and *E. coli.*	[158]

Improvement percentages are calculated by comparing the best performance for each property to the control sample.

**Table 5:** Summary of studies on improving the properties of alginate-based films.

Additives	Properties Improvement	Reference
Aloe vera, garlic oil	**Mechanical properties:** tensile strength increased by 51%, elongation at break increased by 316%; **Water vapor permeability:** 45% reduction; **UV light barrier:** transmittance of UVC, UVB, and UVA decreased by 97%, 96%, and 96%, respectively; **Antimicrobial properties:** *S. aureus*, *E. coli,* and *S. racemosum.*	[125]
Carboxymethyl cellulose, chitosan, CaCl_2_	**Mechanical properties:** tensile strength increased by 81%, elongation at break increased by 46%; **Water vapor permeability:** 4% reduction; **Antimicrobial properties:** *S. aureus* and *E. coli.*	[198]
Cellulose nanofibers, peanut red skin extract	**Mechanical property:** tensile strength increased by 1033%; **Antioxidant property:** the maximum ABTS scavenging activity was 99.28%; **Antibacterial properties:** *E. coli*, *S. typhimurium*, *S. aureus,* and *L. monocytogenes*.	[199]
Cellulose nanowhisker, copper oxide nanoparticles	**Antimicrobial properties:** *S. aureus*, *E. coli*, *Salmonella* spp., *C. albicans*, *Trichoderma* spp.; **Antioxidant property:** DPPH scavenging increased by 41.55%.	[200]
Cu-deposited graphitic carbon nitride (g-C_3_N_4_) nanoparticles, starch	**Mechanical property**: tensile strength increased by 55%; **Water vapor permeability:** 59% reduction; **Antimicrobial properties:** *S. aureus* and *E. coli*.	[201]
Gelatin, aqueous beetroot peel extract	**Mechanical properties:** tensile strength increased by 158%, elongation at break increased by 49%; **Antimicrobial properties:** *S. aureus*, *L. monocytogenes*, *S. enterica,* and *E. coli*; **Antioxidant property:** DPPH scavenging ability increased by 133%.	[202]
Gelatin, green tea extract	**Mechanical property:** tensile strength increased; **Water vapor permeability:** 35% reduction; **Oxygen barrier:** oxygen permeability reduced by 58%; **UV light barrier**; **Antimicrobial properties:** *S. aureus* and *E. coli*.	[203]
Pectin, cinnamic acid	**Mechanical properties:** tensile strength increased by 9%, elongation at break increased by 13%; **Antimicrobial activity:** *B. subtilis*, *MRSA*, *S. aureus*, *Proteus mirabilis* (*P. mirabilis*), *S. typhimurium*, *E. coli*, *A. anitratus*, *Yersinia enterocolitica* (*Y. enterocolitica*), and *Pseudomonas aeruginosa* (*P. aeruginosa*).	[204]
Sulfur nanoparticles	**Mechanical properties:** tensile strength increased by 12%, elastic modulus increased by 107%; **Water vapor permeability:** 41% reduction; **UV light barrier**; **Antibacterial properties:** *E. coli* and *L. monocytogenes.*	[205]
Thymol	**Mechanical properties:** tensile strength increased by 15%, elongation at break increased by 111%; **Water vapor permeability:** 17% reduction; **Water solubility:** 60% reduction; **Antibacterial properties:** *S. aureus* and *E. coli*;.	[206]
Tannic acid	**Water vapor permeability:** 56% reduction; **UV barrier;** **Antioxidant property:** DPPH radical scavenging activity increased from 0 to 89.2%.	[207]

Improvement percentages are calculated by comparing the best performance for each property to the control sample.
